# Network analysis of farmed Atlantic salmon movements in British Columbia, Canada

**DOI:** 10.3389/fvets.2025.1568484

**Published:** 2025-06-18

**Authors:** Ahsan Raquib, K. Larry Hammell, Javier Sanchez, Nicole O’Brien, Krishna Kumar Thakur

**Affiliations:** ^1^Department of Health Management, Atlantic Veterinary College, University of Prince Edward Island, Charlottetown, PE, Canada; ^2^Centre for Veterinary Epidemiological Research (CVER), Atlantic Veterinary College, University of Prince Edward Island, Charlottetown, PE, Canada; ^3^Department of Fisheries, Forestry and Agriculture, Aquatic Animal Health Division, St. John's, NL, Canada

**Keywords:** Atlantic salmon, disease control, fish diseases, network analysis, risk-based surveillance

## Abstract

An inherent issue to the Atlantic salmon aquaculture production is the possible transmission of infectious pathogens due to the transportation of live fish. This study employed network analysis to model the contribution of Atlantic salmon transfers to the spread of pathogens. We used a publicly available salmon transfer dataset covering the period 2015–2022. Official records showed that 812 transfers of Atlantic salmon occurred between various British Columbian (BC) salmon production units in that timeframe. For the purpose of evaluating changes in the network structure of farmed Atlantic salmon movements, the daily networks were aggregated into two-year periods to generate a time-ordered series of biennial movements. The freshwater hatchery and marine netpen sites comprised the two types of facilities that made up the Atlantic salmon transfer network, which consisted of 99 nodes (facilities) and 350 edges (links) overall. All the networks showed both scale-free and small-world topology, which would encourage the persistence and spread of pathogens in the Atlantic salmon facilities while simultaneously making it easier to develop risk-based surveillance techniques by focusing on high centrality nodes. Additionally, the rare occurrence of high betweenness and reach, presence of disassortative mixing, negative correlation between the in- and out-degree and between ingoing and outgoing infection chain of facilities, and the identification of freshwater hatcheries as potential superspreaders all suggest that Atlantic salmon transfers might not play a significant role in the spread of pathogens between facilities in the British Columbian Atlantic salmon farming industry. Community detection revealed two or three communities persistently in the aquaculture management unit (AMU) level network, and it would be more effective to make zoning based on AMU. In conclusion, targeted surveillance efforts on high-centrality facilities can be employed to combat any infectious outbreak in the BC Atlantic salmon industry caused by live Atlantic salmon movement.

## 1 Introduction

Canada’s production volume generated by the salmon farming sector is the fourth largest in the world (1). Salmon is farmed on both the East and West coasts of Canada with about 60% from the province of British Columbia (BC) on the West Coast (2). Several infectious and noninfectious diseases affect salmon production, leading to varying fish mortality and significant economic loss (3).

The local fish movements associated with husbandry and involving an infected, often undetected, farm is a likely factor in pathogen transmission between salmon farms (4, 5). Numerous undocumented and difficult to quantify risk factors contribute to the local spread of infectious diseases, including the passive movement of infected water or organic matter, contact with contaminated personnel or shared equipment, and exposure to infected wild organisms. However, it is also possible that pathogen exposure can occur over long distances through contact with infected (dead or alive) fish moved for production purposes, such as transport to processing plants at harvest, or through contact with contaminated well boats or other maritime vehicles (6, 7).

Typical wild salmon populations start as eggs in freshwater, experience early life stages in freshwater streams, migrate in the spring as smolts to saltwater, and then can travel great distances in the ocean before migrating back to the freshwater stream to spawn. This anadromous life cycle is generally replicated in salmon farming production, spawning, egg incubation and hatching in freshwater, fry and parr production in freshwater hatcheries, transfer of smolts to marine cages then grow-out to harvest. The eggs are incubated and nurtured at the freshwater hatchery, going through the fry and parr stages before they are ready to smoltify. The smoltification physiological and metabolic changes are induced by photoperiod (8–10) and are size-dependent, generally any individual greater than 50 grams is considered capable of adapting to seawater. Fish are transferred to marine grow-out sites until they are harvest size of 4 kg or greater which usually takes 14–24 months (longer cycles will harvest larger fish with the intent to have year-round harvests). Market-sized fish are slaughtered at marine sites and then transported to a processing facility after percussive stunning and partial exsanguination. Salmon farm companies have genetic selection programs with initial selection occurring prior to leaving the freshwater hatchery and they are then maintained at land-based broodstock facilities to avoid exposure to marine pathogens. Broodstock can also be maintained in marine netpen sites, and when this occurs, movements are required to transfer broodstock to freshwater hatcheries. The structure of fish movements in the salmonid industry is pyramidal, with most movements occurring from a few freshwater hatcheries (top) to more marine netpen sites (bottom), and fewer movements occurring between marine netpen sites. During a production cycle, other types of movements are possible, such as the infrequent movement of fish from one marine production site to another, the removal of the dead fish, the delivery of food, and the transportation of equipment and workers (11).

Due to the freshwater and marine environment stages, the production cycle for salmon farming requires fish movements, which has been identified as a risk factor for introducing and spreading infectious fish pathogens (6). For instance, the outbreak of infectious salmon anaemia virus (ISAV) in Scotland (1998–1999) and in Chile (2007–2009) were primarily attributed to such movements (6, 12). Fish movements have also contributed to the transmission of other pathogens, including viral haemorrhagic septicaemia (VHS) (13), bacterial kidney disease (BKD) (14), and infectious pancreatic necrosis virus (IPNV) (15). Some pathogens are infectious only in specific environments or life stages. For example, ISAV is primarily a clinical disease in seawater, but fish may get exposed, including to HPR0, which can later mutate, during their earlier life stage in freshwater. However, this virus has never been reported as present in British Columbia, Canada (16). In contrast, IPNV and BKD affect salmonids in both freshwater and marine environments. Both diseases initially emerged in freshwater before being detected in marine netpen sites, with IPNV typically causing disease in fry and shortly after seawater transfer, while BKD can affect fish across all age groups (17–19). Similarly, *Aeromonas salmonicida*, which causes furunculosis, can affect fish in both freshwater and seawater environments (20, 21). Other examples include *Yersinia ruckeri*, the causative agent of enteric redmouth disease (ERM), which primarily infects fish in freshwater but can cause disease post-transfer in seawater due to prior exposure (22, 23). In light of this risk, characterizing movements will inform plans for production (24) and disease control (25, 26) decisions. Network analysis identifies and quantifies connections between different farms due to movements of things, living or inanimate. This study examined the movement of live fish as an important factor in the spread of pathogens. It provides a standardized statistical summary of the properties of a set of farms and the edges linking them. This method identifies specific farms with greater local and regional connectivity and spatial connection patterns. As a result, it is a valuable tool for locating production units that potentially pose higher risk of contracting or spreading infectious diseases.

Atlantic salmon (*Salmo salar*) is the major farmed salmon species in BC, accounting for more than 90% of the farmed salmon in the province (27, 28). This is the first study aimed (1) to characterize the network of live Atlantic salmon movements and (2) to model the possible influence of each type of facility on the spread of infectious fish pathogens in farmed Atlantic salmon population in BC, Canada.

## 2 Methods

### 2.1 Fish movement data

Data on salmon (Atlantic and Chinook) fish transfers between different fish holdings (freshwater hatcheries or marine netpen sites) in British Columbia, Canada, are recorded and made publicly available on an electronic database on the Government of Canada website known as Open Canada^1^ (29). According to section 56 of the Fishery regulations, authorization from the Department of Fisheries and Oceans (DFO) is required for all fish transfers involving salmon aquaculture in British Columbia, Canada. A signed veterinarian attestation that describes the condition of the fish to be transferred and attests to their health must be included with every transfer authorization application. DFO evaluates these applications to see if the movement could have a negative impact on nearby aquatic species and habitats.

Fish movement records include live salmon movements between separate facilities that have occurred within or between different aquaculture management units of British Columbia, Canada, from 2015 to 2022. The data includes facility type (freshwater hatchery or marine netpen sites) (Table 1), facility name, facility location (latitude and longitude), the unique ID number of the facility, species of salmon transferred, life stage (fry, smolt, adult, brood) (Table 1) of salmon when they were moved, and name of the farming company(ies) involved in the fish transfer.

**Table 1:** Definition of facility types and life stages of Atlantic salmon used in the current Atlantic salmon transfer network.

Facility / Life stage	Definition
Facility type	
Freshwater hatchery	A large freshwater land facility used for growing juvenile Atlantic salmon from eggs to smolts.
Marine netpen sites	Sites with open-water nets in the ocean called “marine netpen” are used to raise Atlantic salmon until they are ready to be harvested.
Life stages	
Fry	A freshwater life stage in which juvenile Atlantic salmon swim freely. After consuming their yolk sac, juvenile salmon (alevins) become fry.
Smolts	A transitional phase in the development of juvenile Atlantic salmon during which they experience physiological changes that enable them to adjust to life in saltwater.
Adults	Atlantic salmon at their marine life stage.
Brood	Adult Atlantic salmon are utilized as “parent fish.” These large fish are raised to maturity, even past the time of harvest. The next generation of farmed Atlantic salmon is raised using their eggs and milt.

### 2.2 Data cleaning

There were 848 fish transfers recorded between 2015 to 2022. One transfer occurred within a research facility, which was removed. The majority (95.75%) of transfers involved the movement of Atlantic salmon, and the rest (*n* = 36) involved Chinook salmon. Since the objectives of this study were restricted to Atlantic Salmon transfer network in British Columbia, Canada, records of Chinook Salmon movements were removed from further analysis.

### 2.3 Network analysis

“iGraph” package of R software version 4.2.2 was used for conducting network analysis (30). Additionally, the EpiContactTrace package was used to calculate the ingoing and outgoing infection chain (31). Network parameters calculated for facility-level Atlantic salmon transfer networks are detailed in Table 2. The network of the Atlantic salmon production facilities was represented as a temporally directed graph with the notation G = (V, E), where V was a group of nodes (facility), and E was a set of directed edges that indicated movements in the network. To examine the overall facility-level Atlantic salmon movement, we employed a static version of the directed network with edges aggregated over 8 years (2015–2022). As the production cycle for Atlantic salmon lasts around 2 years (32), we created a time-ordered series of biennial snapshots by aggregating the daily networks into two-year periods, to investigate notable changes in the network structure of the Atlantic salmon transfers from 2015 to 2022.

**Table 2:** Definition of network metrics used for assessing movement network of Atlantic salmon in British Columbia, Canada, from 2015 to 2022.

Network metrics	Range	Definition
Nodes	0 to ∞	Here, nodes are either freshwater hatcheries or marine netpen sites between which Atlantic salmon transfer networks are being studied (57).
Edges	0 to ∞	It is the direct link between either freshwater hatcheries or marine netpen sites (57).
Assortativity (degree)	−1 to 1	It measures the proportion of connections between nodes with similar degrees, and its value ranges between −1 and +1. A negative value would mean nodes (freshwater hatcheries or marine netpen sites) are linked with other nodes with dissimilar attributes (58, 59).
Network diameter	0 to ∞	It is the longest shortest distance between any two epidemiological units. A disease will take fewer generations to spread throughout the network if the diameter is smaller (57).
Network density	0 to 1	Shows the proportion of actual transfers among all the Atlantic salmon facilities (freshwater hatcheries or marine netpen sites) (57).
Reciprocity	0 to 1	Percentage of edges with mutual connections (A measure of the likelihood that two vertex pairs will form a link with one another) (50)
Weekly connected components (WCC)	0 to ∞	A portion of facilities where, regardless of the direction of Atlantic salmon movement, there is a link between every pair of facilities. Calculating the higher bound of the maximal epidemic size is possible using the size of the WCC (60).
Strongly connected components (SCC)	0 to ∞	A portion of an Atlantic salmon transfer network where all facilities are accessible by following the network’s transfer linkages. Calculating the lower bound of the maximal epidemic size is possible using the size of the largest SCC (60).
Network Reach	0 to ∞	The number of freshwater hatcheries or marine netpen sites that can be reached by taking directed pathways starting at the freshwater hatchery or marine netpen site. It calculates a facility’s ability to disseminate a disease throughout the network (39, 43).
Centrality parameter	–	The centrality parameter, which includes betweenness, infection chain and degree distributions, determines the importance and role of each facility within the network (57).
Total-degree	0 to ∞	The sum of all transfers to or from a freshwater hatchery or marine netpen site (61).
In-degree	0 to ∞	Number of incoming transfers to a freshwater hatchery or marine netpen site (57).
Out-degree	0 to ∞	Number of outgoing transfers from a freshwater hatchery or marine netpen site (57).
Superspreader	–	Freshwater hatcheries or marine netpen sites with out-degree greater than or equal to the 95th percentile.
Supersink	–	Freshwater hatcheries or marine netpen sites with in-degree greater than or equal to the 95th percentile.
Hotspot	–	Freshwater hatcheries or marine netpen sites meeting both the superspreader and super sink criteria.
Ingoing infection chain	0 to ∞	The count of both direct and indirect trade interactions, accounting for the chronological order of the contacts leading to a particular facility (62).
Outgoing infection chain	0 to ∞	Number of trade interactions, both direct and indirect, that start at a particular facility while accounting for the contact’s chronological order (63, 64).
Node betweenness	0 to ∞	The number of shortest routes between nodes i and j that pass-through node k reveals the node’s capacity to spread an infection inside the network. Removing an epidemiological unit with high betweenness from a network will help control the spread of disease in the network (65).
Average path length	0 to ∞	The shortest path is the minimum number of arcs from one node to another node. Average path length measures the mean number of steps required for an infection to travel from a random epidemiological unit to another arbitrary epidemiological unit (66).
Clustering coefficient (CC)	0 to 1	Measure the percentage of all potential triplets with three nodes connected and form a closed triangle. The infection spreads rapidly in a network with high clustering coefficient (57, 67).
Random network	–	A network where each edge is independent of the others (33)
Small world network	–	A network having a small path length and a high clustering coefficient (66).
Scale-free network	–	Network with a power-law distribution of degrees (68).

Within the biennial and overall facility-level networks, we classified facilities as follows: “superspreaders,” “super-sinks,” and “hotspots” (meeting both the superspreader and super sink criteria). Facilities with in-degree and out-degree equal to or above the 95th percentile were used to establish the out-degree and in-degree thresholds for classifying “superspreaders” and “super-sinks” respectively.

We assessed the mean node and edge persistence to evaluate the consistency in Atlantic salmon facility-level transfer networks from 2015 to 2022. To do this, the number of nodes or edges in all four biennial network windows was divided separately by the geometric means of those counts (17).

The facility-level networks had two types of nodes: freshwater hatchery and marine netpen sites. Box plots were used to visualize the median, minimum, and maximum values, the 25th and 75th percentiles for each of the centrality measures by the type of nodes (facilities). To investigate the relationships between the centrality parameters “degree,” “infection chain, “and “node betweenness,” we performed a Spearman rank correlation. A heatmap was used to graphically display these Spearman rank correlations.

The properties of small-world topology, characterized by strong clustering and short path length, were also examined in all four biennial and overall facility-level networks. By making sure that the facility-level networks and random networks have the same number of nodes and edges, we were able to compare the clustering coefficient (CC) and average path length (APL) of facility-level networks with those of 1,000 randomly generated networks (RN) created using the Erdos-Renyi model (33). The small-world-ness (S) of Atlantic salmon transfer networks (ASN) was assessed by following the Equation 1 (34–36):


(1) $$S=({\mathrm{CC}}_{\mathrm{ASN}}/{\mathrm{CC}}_{\mathrm{RN}})/({\mathrm{APL}}_{\mathrm{ASN}}/{\mathrm{APL}}_{\mathrm{RN}})$$

If networks had S > 1, they were said to have a small-world topology. We used the R package poweRlaw (version 0.70.6) to examine whether the facility-level network of Atlantic salmon transfer exhibited scale-free characteristics, characterized by a highly diverse degree distribution that follows a power law distribution. Utilizing parameters derived through Maximum Likelihood estimation, we fitted the network’s degree distribution to a power law model and evaluated the fit using the Kolmogorov-Smirnoff test (37, 38).

In a static network, some paths may appear between nodes that aren’t actually possible when we consider the order of events over time. These paths can ignore the true sequence of interactions and therefore do not exist in the corresponding temporal network. To assess how accurately a static network represents the real, time-ordered structure, Lentz et al. (39) introduced a metric called causal fidelity. This measure compares the number of valid paths in the temporal network that are also present in the static version. To calculate this, accessibility matrices were constructed for both the static and temporal versions of each biennial and the overall network, using a one-day temporal resolution to capture which nodes can realistically reach others over time. The Equation 2 provided below was used to assess causal fidelity (C):


(2) $$C=(\begin{array}{l} \text{Number of paths in}\;\\ \text{temporal network} \end{array})/(\begin{array}{l} \text{Number of paths}\;\\ \text{in static network} \end{array})$$

A causal fidelity value of 1 indicates that the static network accurately captures the time-respecting paths of the temporal network. In contrast, when causal fidelity is close to 0, it indicates that the static network fails to capture the true sequence of interactions. The causal error, calculated as the reciprocal of causal fidelity (1/C), provides an estimate of the degree to which a static network may overestimate the potential extent of a disease outbreak.

The network’s susceptibility to targeted node removal was assessed using percolation analysis. Using this method, nodes were systematically removed one at a time, taking into account their relative rankings in different centrality metrics, and the effects were monitored on the size of the largest weakly connected component (LWCC) across all facility-level Atlantic salmon transfer networks at each stage of node removal. This analysis was designed to simulate the effects of real-world interventions, such as trade restrictions, quarantine, vaccination, fallowing, culling or increased fish testing on disease spread through the Atlantic salmon transfer networks. In this context, the removal of a node represents the exclusion of a facility from the disease transmission network, which could possibly be done by any one or combination of these intervention strategies. In-degree, out-degree, ingoing infection chains, outgoing infection chains, and betweenness were the centrality metrics that guided the process of removing nodes. A comparison was made between the network’s structural sensitivity to the purposeful removal of central nodes and the random removal of nodes in order to better evaluate the impact of focused interventions on network connectivity.

Finally, an aquaculture management unit (AMU) level network was also created, aggregating all nodes on the same AMU and identified by the AMU name, and movements (edges) within AMU were removed while edges connecting AMUs to unique AMUs were amalgamated based on their directionality to represent connectivity among AMUs. We used the walktrap community detection method (40) to find communities—groups of AMU or facilities that display comparable connectivity patterns. Walktrap works on the notion that, in comparison to nodes in other communities, nodes within the same community often have more connections and shorter random walks between them (40).

## 3 Results

### 3.1 Data description

**Figure 1:**
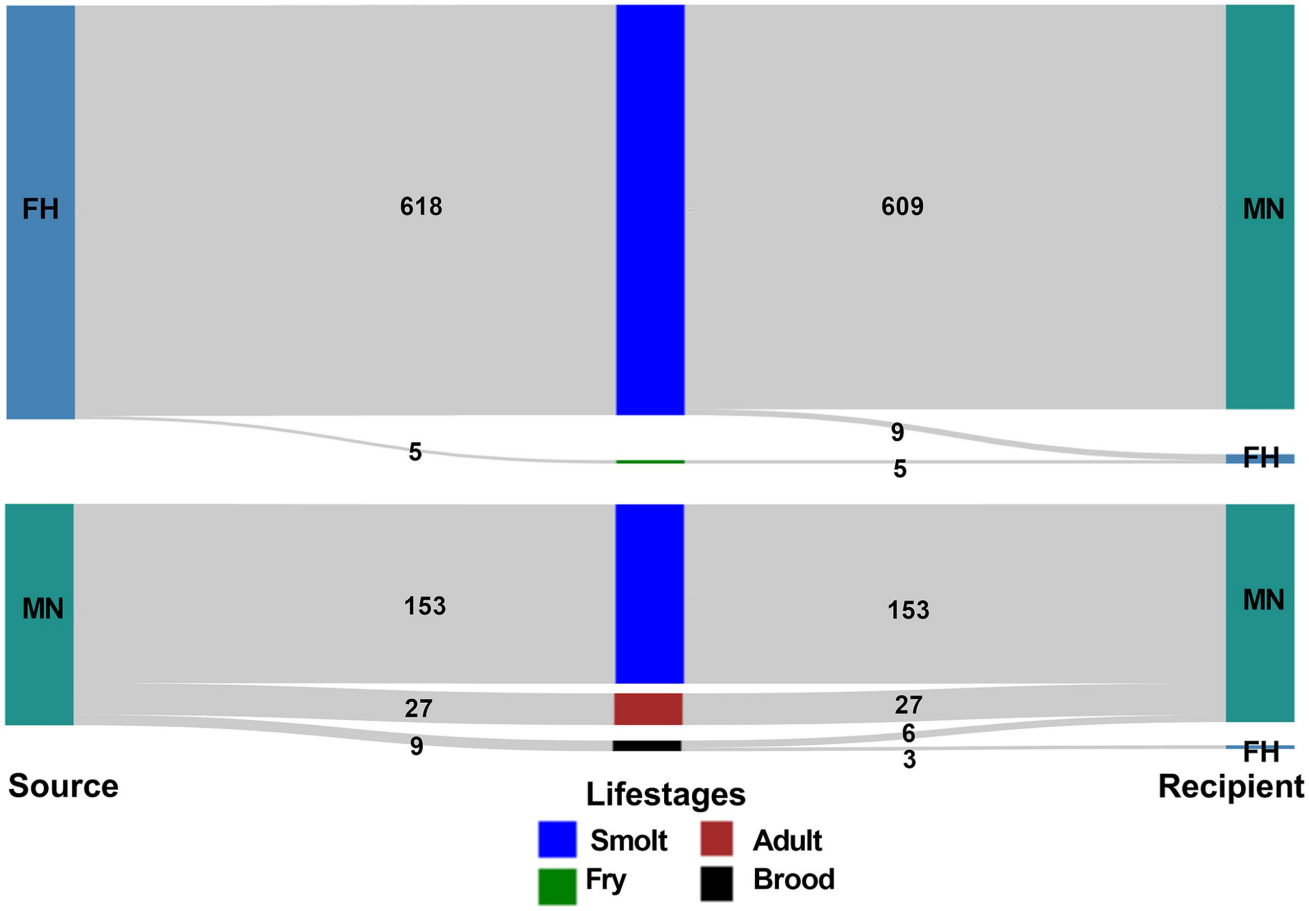
Visualization of the frequency of Atlantic salmon transfers between different facility types (FH-freshwater hatchery, MN-marine netpen site) from 2015 to 2022 in BC, Canada.

From 2015 to 2022, 11 freshwater hatcheries and 88 marine netpen sites (99 facilities in total) (Supplementary Table 1) were involved in the movements of live Atlantic salmon in British Columbia, and a total of 812 transfers were recorded during that period (Table 3). The highest number (*n* = 234) of transfers occurred during 2021–22, though the lowest numbers (*n* = 66) of facilities were involved in that biennial window (Supplementary Table 2). Most (*n* = 609) of the transfers involved movement of Atlantic salmon smolts from freshwater hatcheries to marine netpen sites. Moreover, there were 153 transfers of smolts from marine netpen sites to marine netpen sites. Figure 1 depicts all the movements reported in the database.

**Table 3:** Overall movement of atlantic salmon at different life stages in British Columbia, Canada, from 2015 to 2022.

Category	Number of facilities (nodes)	Number of edges (unique link)	Number of transfers (total link)
Overall	99	350	812
Life stages			
Fry	4	3	5
Smolt	98	329	771
Adult	20	17	27
Brood	11	9	9

Marine netpen sites were on the receiving end of the transfers most of the time from freshwater hatcheries and other marine netpen sites in all biennial networks evaluated. Freshwater hatcheries rarely received Atlantic salmon transfers (*n* = 17) during the study period, 3 of these transfers (all in the 2021–22 biennial window) were recorded as originating at marine netpen sites, and the remaining 14 transfers originated from freshwater hatcheries (Supplementary Table 3).

**Figure 2:**
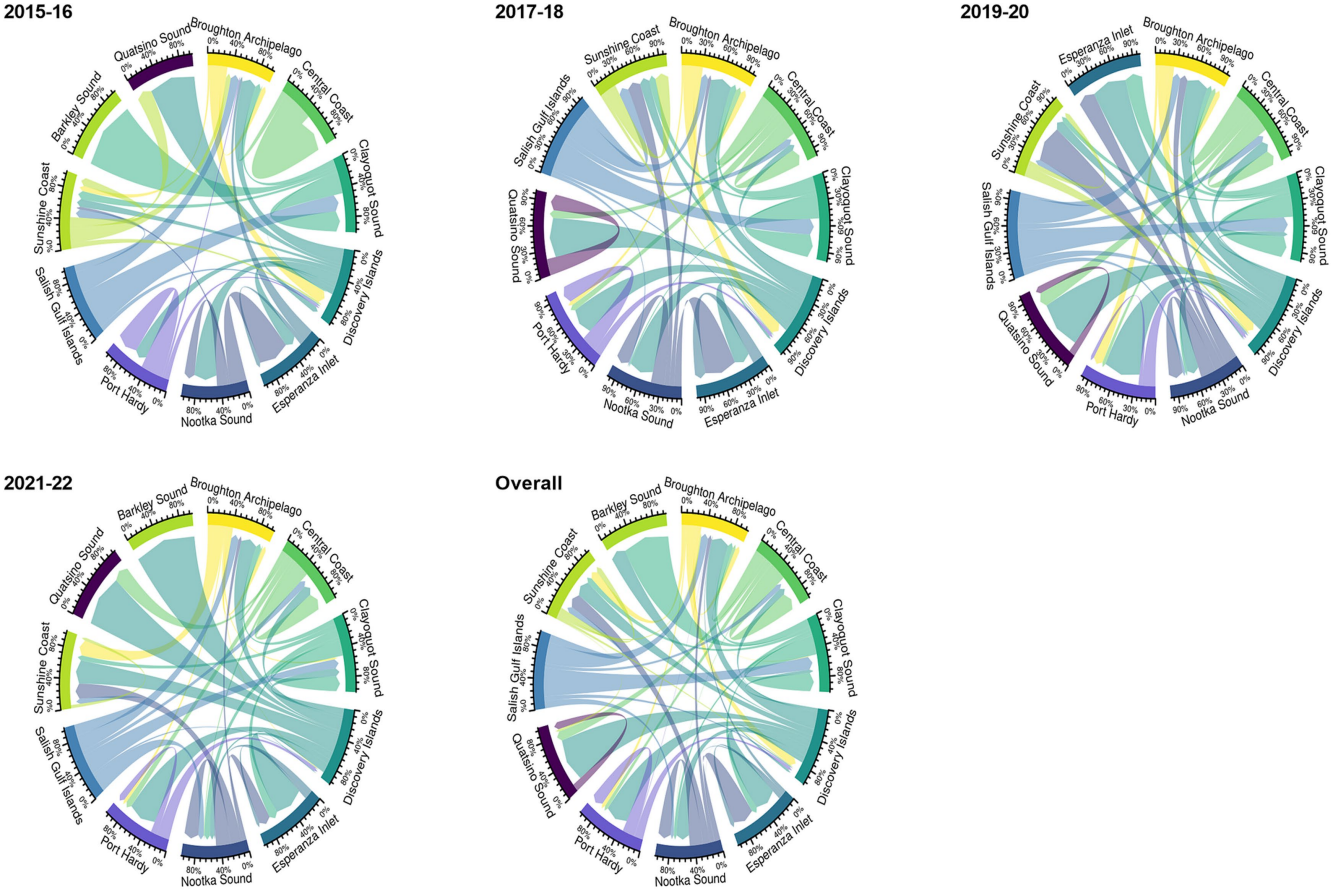
The proportion of intra-and inter-aquaculture management unit (AMU) Atlantic salmon transfer flows in BC, Canada, from 2015 to 2022 (biennial and overall network) is shown by edge bundling. The weight of the arrows reflects the frequency of transfer between AMU. While inter-AMU movement is represented by arrows pointing to various AMU, intra-AMU movement is represented by arrows starting and ending in the same AMU. Each color represents a specific AMU.

Among the 11 aquaculture management units (AMU) in overall network, facilities in Discovery Islands and Broughton Archipelago supplied (*n* = 267) and received (*n* = 197) fish transfers for the highest number of times, respectively. Facilities in Barkley Sound (only 1 facility) never supplied any fish; on the other hand, facilities in the Salish Gulf Islands were never on the receiving end of the transfers (Figure 2). Inter-AMU fish transfers (75.25%) were more frequent than intra-AMU transfers (24.75%).

### 3.2 Network properties

The network properties of overall and biennial Atlantic salmon transfer networks are presented in Table 4. The first three biennial networks (2015–16, 2017–18, and 2019–20) had positive assortativity (0.04 to 0.14); however, the 2021–22 network had a negative assortativity of−0.07. Overall, the network had a disassortative degree of −0.10, which means nodes exhibited a slight tendency to link with nodes with dissimilar attributes (facilities with high out-degree were linked with low in-degree facilities or vice-versa). The network diameter (3 to 4) and density (0.02 to 0.04) were small and were stable over all the network snapshots. Mutual connections (reciprocity) were absent during the first three network snapshots and was only 0.05 in the 2021–22 biennial network. Overall, reciprocity for the network was 0.04, indicating that only 4% of links between facilities had bidirectional transfers.

**Table 4:** Network properties for movement of Atlantic salmon biennial and overall networks in British Columbia, Canada, from 2015 to 2022.

Network properties		2015–16	2017–18	2019–20	2021–22	Overall
Number of nodes		73	85	78	66	99
Number of edges		118	153	150	165	350
Assortativity (degree)		0.04	0.04	0.14	-0.07	−0.10
Average shortest path length		1.28	1.58	1.33	1.61	1.62
Network diameter		3	4	3	4	4
Network density		0.02	0.02	0.03	0.04	0.04
Reciprocity		0	0	0	0.05	0.04
In-degree	Mean	1.62	1.8	1.92	2.52	3.54
	Median	2	1	2	2	3
	Minimum	0	0	0	0	0
	95%	3.4	4.8	4	6	7
	Maximum	5	6	5	8	10
Out-degree	Mean	1.62	1.8	1.92	2.52	3.54
	Median	0	0	0	0	1
	Minimum	0	0	0	0	0
	95%	8.4	12	14.15	14.75	22.1
	Maximum	17	17	19	26	41
Total-degree	Mean	3.23	3.6	3.85	5.03	7.07
	Median	2	2	2.5	3	5
	Minimum	1	1	1	1	1
	95%	8.4	12	14.15	17.25	23.2
	Maximum	17	17	19	26	41
Ingoing infection chain	Mean	2	2.98	2.5	3.62	5.38
	Median	2	2	2	3	4
	Minimum	0	0	0	0	0
	95%	5	7	5	8	11
	Maximum	7	13	6	13	22
Outgoing infection chain	Mean	2	2.98	2.5	3.62	5.38
	Median	0	0	0	0	1
	Minimum	0	0	0	0	0
	95%	9.4	20	17.3	19	37.1
	Maximum	18	29	28	30	45
Node betweenness	Mean	0.60	1.93	0.88	2.86	4.15
	Median	0	0	0	0	0
	Minimum	0	0	0	0	0
	95%	5	12.6	4.23	9.75	18.30
	Maximum	7	37	25	82	91
Node reach	Mean	3.16	4.34	3.72	5.68	7.65
	Median	1	1	1	1	3
	Minimum	1	1	1	1	1
	95%	12.4	23.6	19.6	23.25	41.3
	Maximum	20	36	30	35	51
Clustering coefficient		0.09	0.09	0.11	0.14	0.19
Size of largest strongly connected components (LSCC)		1	1	1	5	6
Proportion of LSCC (% of nodes)		0.01	0.01	0.01	0.08	0.06
Size of largest weakly connected components (LWCC)		35	42	41	64	97
Proportion of LWCC (% of nodes)		0.48	0.49	0.53	0.97	0.98

Overall, the Atlantic salmon transfer network had 2 weakly connected components (WCC) and 88 strongly connected components (SCC) with a maximum length comprising 97 (98% of network size) and 6 (6% of the network size) nodes, respectively (Supplementary Table 4). The maximum length of WCC (*n* = 64) and SCC (*n* = 5) was observed in the 2021–2022 biennial network. During 2015–22, the Atlantic salmon transfer network appeared to have a multimodal reach distribution, where 63 nodes had a network reach of more than 1. Only one freshwater hatchery had a reach of less than 20, but no marine netpen sites had a reach of more than 20 (Supplementary Figure 1).

**Figure 3:**
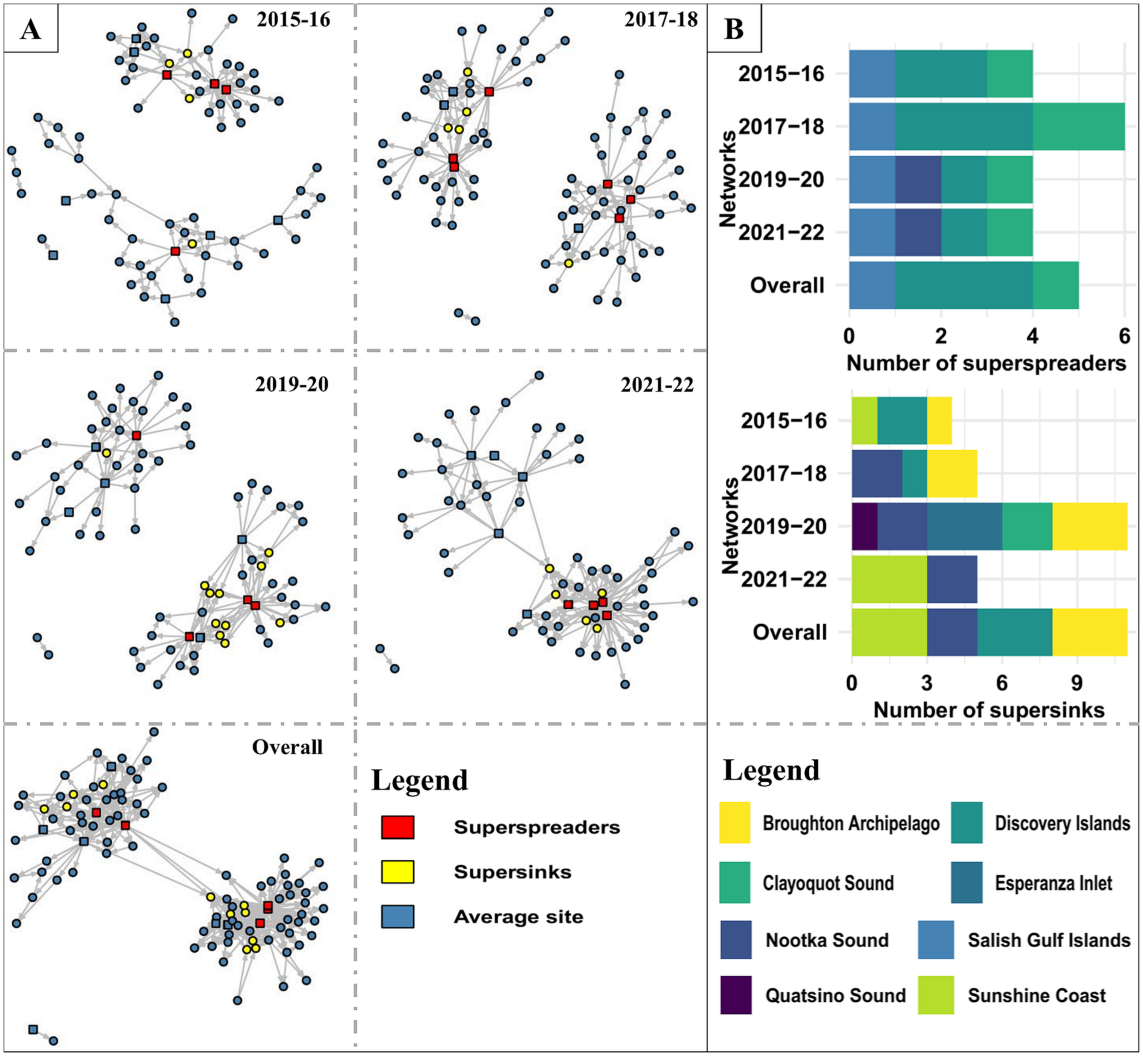
**(A)** Visualization of the Atlantic salmon transfer networks (biennial and overall network) in BC, Canada. Circles and squares represent marine netpen sites and freshwater hatcheries, respectively. **(B)** Distribution of superspreaders and supersinks in different AMUs.

The facility’s mean degree (total-degree) steadily increased (3.23 to 5.03) over the four biennial networks (Figure 3). Overall, facilities had an average of 7.07 connections (total-degree) to other facilities (range: 1–41), with an average of 3.54 incoming (range: 0–10) and outgoing (range: 0–41) connections. Only a small percentage of facilities were highly linked; around 20% of the facilities held 46 to 53% of the total links in the biennial networks, with the highest and lowest links observed in 2021–22 and 2015–16, respectively. The degree distribution for all facilities included in the Atlantic salmon transfer network, whether it was a biennial or overall network, was rightly skewed. Similar to degrees, the mean infection chain increased from 2 to 3.62 in four biennial networks, with maximum ingoing infection chain in 2017–18 and 2021–22 and maximum outgoing infection chain in 2021–22. We identified 5 (5.05% of the facilities) superspreader facilities and 11 (11.11% of the facilities) super-sink facilities in the overall network (Figure 3). All the superspreaders and super-sinks identified in biennial and overall networks were freshwater hatcheries and marine netpen sites, respectively. No hotspot was found on any of the Atlantic salmon transfer networks in BC. The mean persistence of the node was higher than the edge in the Atlantic salmon transfer network, which was 0.56 and 0.14, respectively.

**Figure 4:**
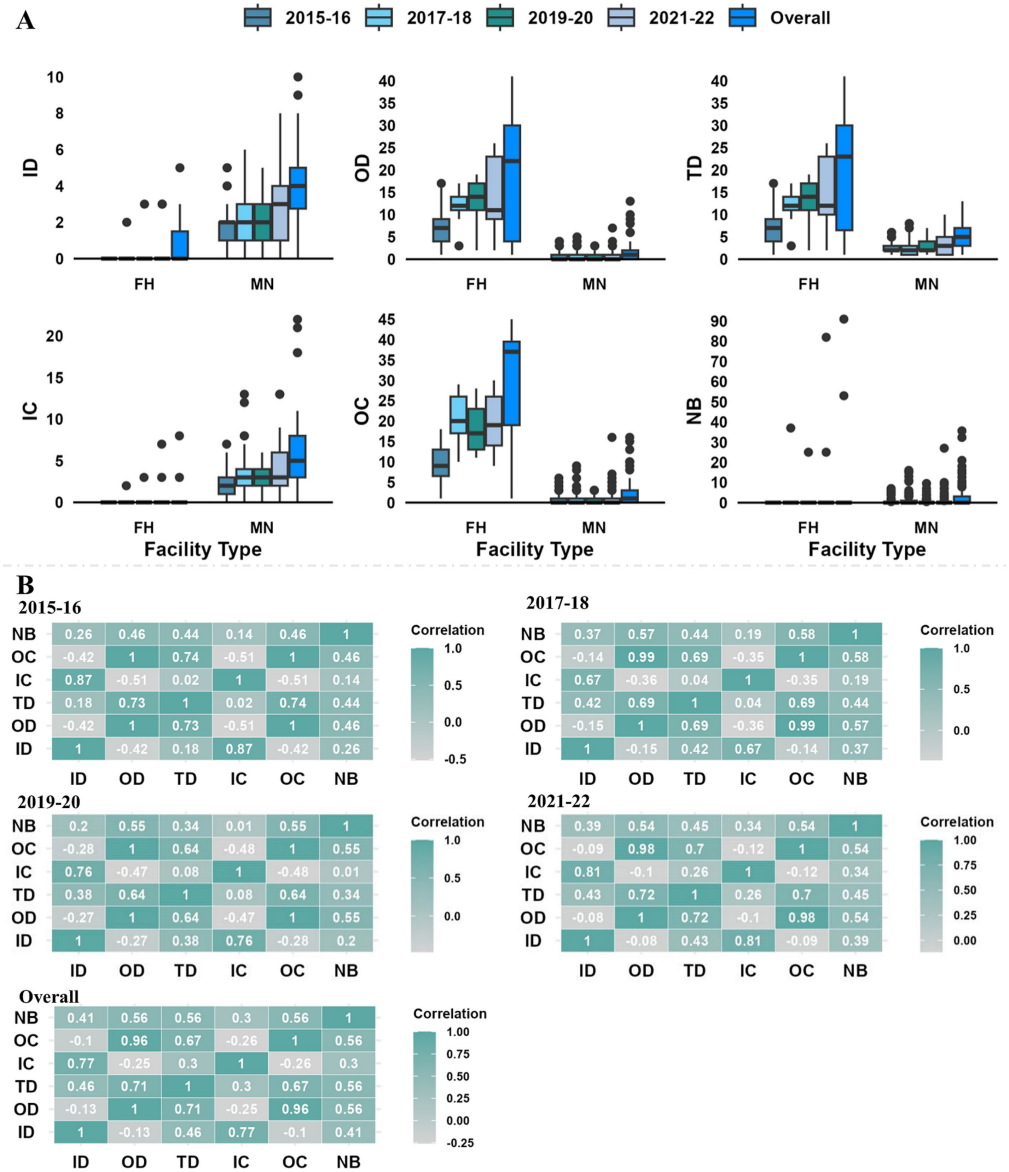
**(A)** Distribution of centrality parameters (ID-in-degree, OD-out-degree, TD-total degree, IC-ingoing infection chain, OC-outgoing infection chain, and NB-node betweenness) in different facility types (FH-freshwater hatcheries, MN-marine netpen site); **(B)** Spearman rank correlation between centrality parameters for the biennial and overall Atlantic salmon transfer networks in BC, Canada.

The Spearman rank correlation (Figure 4B) between centrality parameters in each biennial network revealed a positive correlation among all the centrality parameters except in-degree with out-degree (r = −0.42 to −0.08) and outgoing infection chain (r = −0.42 to −0.09) and out-degree with ingoing infection chain (−0.51 to −0.10). A negative connection between facilities in- and out-degrees indicates that facilities that get fish from a large number of other facilities tend to send fish to a small number of other facilities or vice-versa. However, a highly positive correlation was observed between out-degree and total-degree (r = 0.64 to 0.73), in-degree and ingoing infection chain (r = 0.67 to 0.87), and between out-degree and outgoing infection chain (r = 0.96 to 1).

### 3.3 Small world and scale-free topology

**Figure 5:**
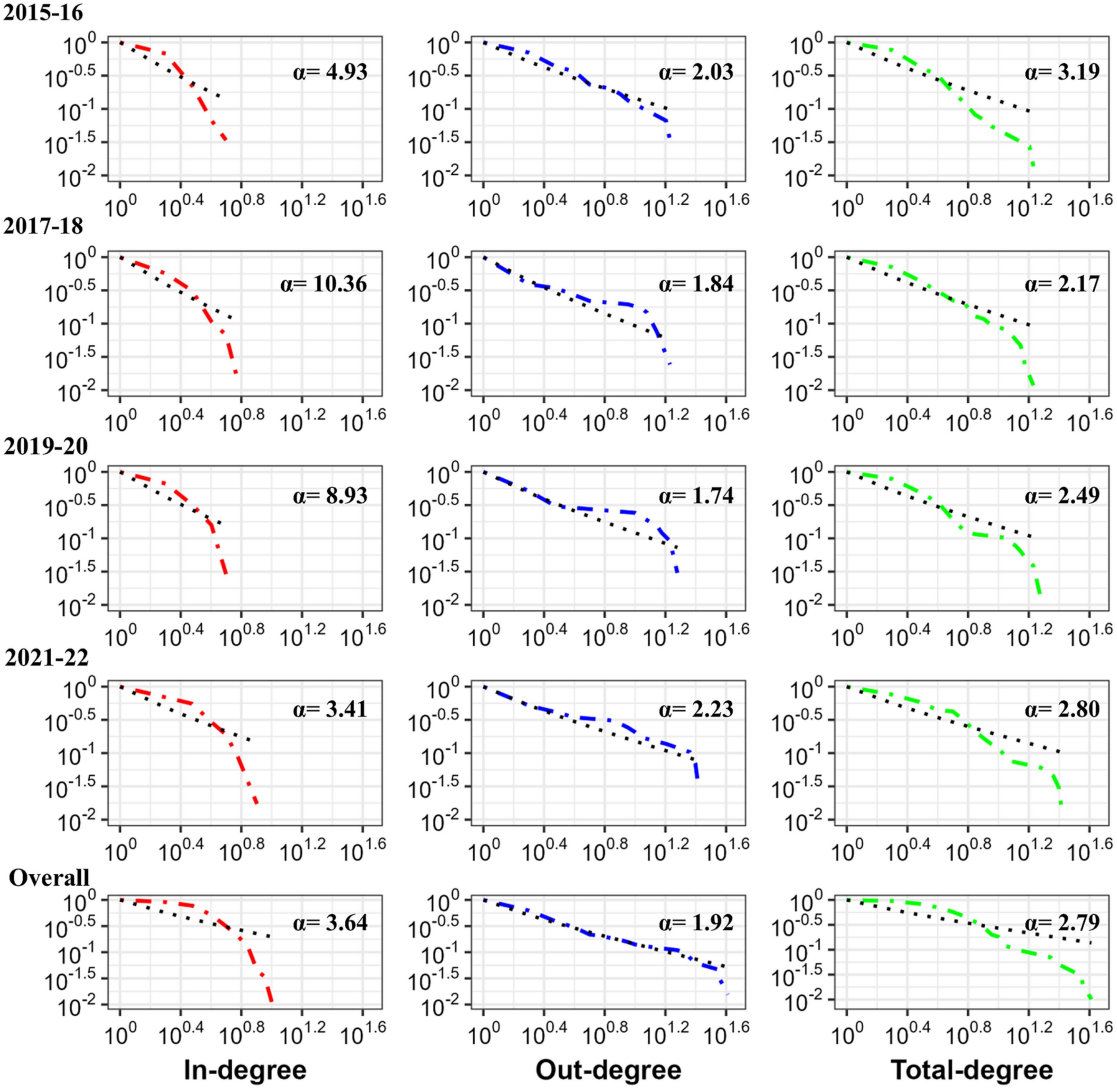
Degree distributions of Atlantic salmon transfer networks (biennial and overall) in BC, Canada. The cumulative frequencies of the node’s degree distributions are shown on a log–log scale. The maximum likelihood approach (38) approximated each degree distribution as a power law (black dashed lines showing power law for degree).

Although the average shortest path length varied in the four biennial networks, it remained low. It would take less than two steps (average shortest path length: 1.28 to 1.62) for an infection to reach any other facility in the overall Atlantic salmon transfer network. Clustering coefficient increased from 0.09 to 0.14 over the 4 biennial networks, with an overall clustering coefficient of 0.19, indicating that facilities were somewhat linked to their neighboring facilities. Random networks (Supplementary Table 5) had higher average path length (2.72 to 3.61) and clustering coefficient varying from 0.04 to 0.08. All the biennial networks and overall network met the criteria (Supplementary Table 5) to be characterized as a small world network. Furthermore, Atlantic salmon transfer network degree (total degree, in-degree, and out-degree) distributions followed a power law distribution (*p* > 0.05) for all four biennial networks and the overall network, which means few nodes with many links constantly affected the network’s dynamics and structure (Figure 5).

### 3.4 Centrality parameters of different facilities

The distribution of centrality parameters of different facility types in the overall and biennial networks is provided in Figure 4A. Only in-degree and ingoing infection chain had higher values (mean, median, and maximum) for marine netpen sites, while other centrality parameters were higher for freshwater hatcheries in all biennial and overall networks. The maximum unique incoming transfer for a freshwater hatchery (e.g., In-degree = 3) was very low, and 82% of the freshwater hatcheries never received any transfer. On the other hand, almost 40% of marine netpen sites never had outgoing movements, with a maximum unique outgoing fish movement of 10 for a marine netpen site.

### 3.5 Causal fidelity

Causal fidelity of all the biennial networks and the overall network was presented in Supplementary Table 6. In all the biennial networks and overall network, causal fidelity was higher than 0.80, with the highest (0.95) in the biennial year 2015–16 and lowest (0.81) in 2021–22. Causal errors across all networks ranged from 1.05 to 1.23, indicating that static representations would overestimate disease outbreak size by 5 to 23%.

### 3.6 Percolation analysis

**Figure 6:**
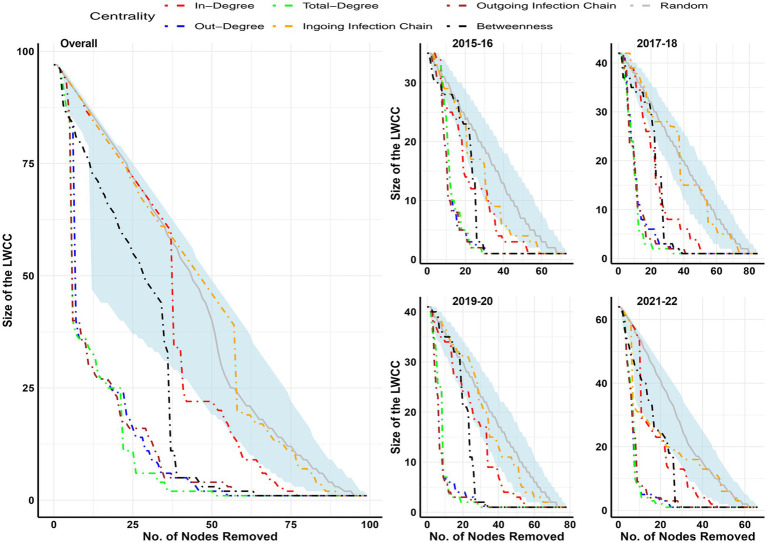
Effectiveness of targeted removal of nodes over the size of LWCC in different biennial and overall networks. Over a thousand simulations, random node removal is represented by the median value (gray line) and its 95 percentile (light blue shaded area).

Percolation analysis results were presented in Figure 6, and the influence of different facility types on the reduction of LWCC was presented in Supplementary Figure 2. The findings indicate that the targeted removal of facilities according to their out-degree, total degree, and outgoing infection chain was the most successful method for reducing the size of the LWCC. About 75% of the size of LWCC was possible to be reduced by the targeted removal of 20% of facilities in all studied networks; additionally, in the biennial networks of 2017–18, 2019–020, and 2021–22, this could be accomplished by removing less than 15% of the facilities. The importance of freshwater hatchery’s contribution to the shrinkage of LWCC was demonstrated by percolation analysis conducted in different types of facilities. It was feasible to lower LWCC by 35–48% by simply eliminating freshwater hatcheries (9 to11) from the network in the biennial networks of 2015–16, 2017–18, and 2021–222 and by more than 65% in the 2019–20 biennial network and overall network. However, if only marine netpen sites were strategically removed, around 44–61% of marine netpen sites would still need to be removed to lower the size of the LWCC by 75%.

### 3.7 Community detection

**Figure 7:**
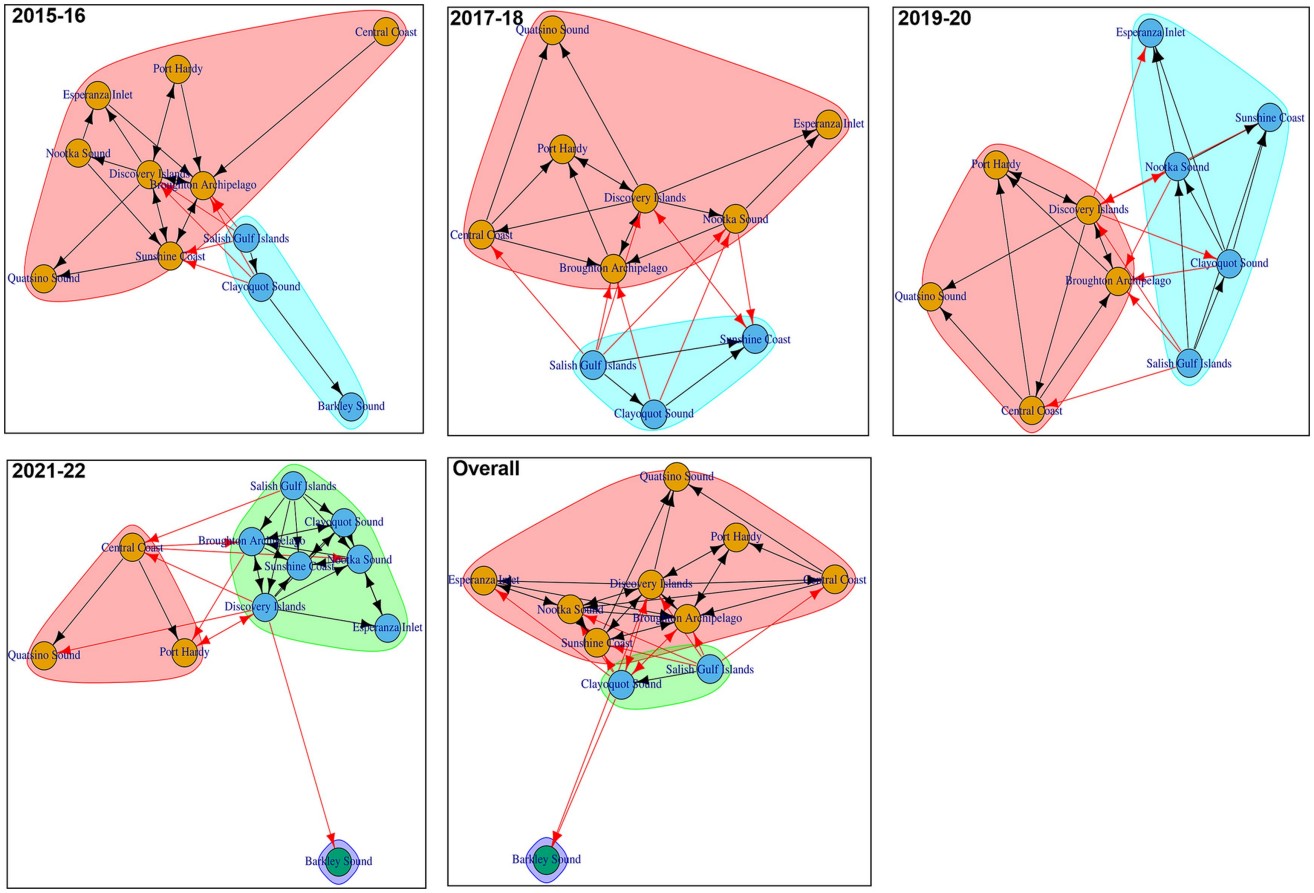
Trade communities in different AMU-level Atlantic salmon transfer networks. Each shaded color represents a specific community within the transfer network. Arrows indicate the direction of transfers between different AMUs (red arrow- transfers between AMUs of different communities, black arrow- transfers between AMUs of same community).

Figure 7 shows the communities detected in the biennial and overall AMU-level networks. Either two or three communities were detected in each biennial or overall AMU-level network. 1 AMU was part of the smallest community (2021–22 biennial network and overall network), while 8 AMU were in the largest community (2015–16 biennial network and overall network). In facility-level biennial networks, we detected 7 to 16 communities (Supplementary Figure 3), with the largest and smallest number of communities in 2021–22 and 2019–20, respectively.

## 4 Discussion

In this study, we conducted a novel investigation of the movement patterns of live-farmed Atlantic salmon within the province of British Columbia, Canada, using network analysis (NA) to shed light on the potential transmission route for infectious diseases. However, NA has previously been used to evaluate the network structure of the salmonid industry in Great Britain (41), England and Wales (42), Scotland (25, 43, 44), and Ireland (45). This study has utilized publicly available data to construct Atlantic salmon transfer networks and offers an epidemiological perspective on this temporal network and applications based on network-based strategies for monitoring and managing infectious diseases.

Our networks are primarily distinguished from those previously explored for Great Britain (41), Scotland (43), England and Wales (42), and Ireland (45) by their size. Only Ireland had fewer nodes and edges, which might be because that study explored a network structure for only 1 year. The mean degrees for the four biennial networks were higher than those of the previously studied salmonoid transfer networks of Ireland (45), Scotland (43), England, and Wales (42). However, the density of our overall (or biennial) network was almost similar to that of Scotland (43) and Ireland (45). The mean degree of the biennial networks is higher than that of the other salmonoid transfer networks, suggesting more connections per node; however, similar density implies that the percentage of potential connections that are real connections stays comparable. Variations in mean degrees may result from differences in the industry’s size, operational scales, regulations, and biosecurity protocols.

The first 3 biennial networks of the current study were positively assortative, then it became disassortative during 2021–2022, which denotes that the structure of the salmon industry changed in terms of connectivity. This might be the result of shifting demographics in the Atlantic salmon industry from 2019 and a decline in the number of facilities at Discovery Island and the Broughton Archipelago. Several marine netpen sites in these two AMUs were closed due to growing concerns over their potential impact on wild salmon populations, particularly regarding the transmission of diseases and sea lice, which may have disrupted historical transfer patterns and contributed to the observed shift in network assortativity (46, 47). These changes are evident in 2021–22 biennial window (Supplementary Table 7), where the number of facilities involved in fish transfer in the Discovery Islands decreased to 6 from 16 in the last two biennial windows, and the number of facilities also decreased in the Broughton Archipelago in that time frame. Identical to the 2021–22 biennial network and overall network, a disassortative network structure was also observed in Ireland’s (45), England’s, and Wales’s (42) salmon transfer networks. Disassortative mixing between facilities resists the spread of infection, resulting in a lower basic reproduction number (R_0_) (43). Moreover, the reduction of the possibility of an epidemic spreading might be aided by the presence of both disassortative mixing and negative correlation between the in-and out-degree of facilities within the Atlantic salmon transfer network. Because high-risk facilities—that is, facilities that get fish from many facilities—are less likely to redistribute fish themselves or to send (potentially infected) fish to facilities with high out-degrees (which would again redistribute infections to other facilities), which would encourage the spread of infections (11, 45, 48). Therefore, facilities with high degrees will disperse across the entire network, which can be vulnerable to targeted attacks on these high-degree facilities to dismantle the network connectivity (49).

All the Atlantic salmon transfer networks had both small world and scale-free characteristics, which implied that networks had short average path lengths and high clustering coefficients and specific facilities consistently had a high level of connectivity, while most other facilities had fewer connections. Thakur et al. (50) pointed out that network topology characterization has important consequences for disease transmission, surveillance, and control strategies. Disease outbreaks can quickly spread inside closely-knit clusters with a small-world topology and connect to distant regions through a finite number of connections. But, compared to random networks, the overall extent of the outbreak is generally less (49). As opposed to random networks, scale-free topologies are predicted to have more widespread outbreaks because hubs serve as the primary sites for disease transmission. When nearby facilities or nodes are infected, disease outbreaks frequently begin to spread rapidly, but once they get to secondary contacts, the transmission rate begins to reduce (51). Furthermore, the power-law fitting of network degrees showed a right-skewed distribution. Such centrality parameter distributions indicate that networks are typically resistant to random node removal. However, if the most central facilities or their connectivity with other facilities are strategically removed, the network structure may be altered and even fragmented. This strategic network breakdown can stop the transmission of pathogens and effectively prevent the spread of infections (52, 53).

According to the centrality metrics of the various facility types, the freshwater hatcheries played a crucial part in our study of Atlantic salmon transfer networks. All the identified superspreaders were freshwater hatcheries. Furthermore, freshwater hatcheries had the highest values for outdegree, outgoing infection chain, and betweenness, which means they can be critical in transmitting infections. However, DFO inspects freshwater hatcheries within 3 months of any proposed transfer, collects samples of recently dead fish, and performs diagnostic tests before authorizing fish transfers. Any fish health concerns need to be addressed prior to any transfers, which leaves minimal risk of transferring pathogens from freshwater hatcheries. Freshwater hatchery fish are younger and have yet to be exposed to marine pathogens, so only vertically transmitted undetected infections or infections arising from freshwater exposures are of concern. Industry biosecurity and surveillance programs, as well as government inspections, reduce the probability of either of these occurring, thus further minimizing the risk of pathogen transfer through freshwater hatcheries. Although unlikely for reasons already stated, introducing any new variant of viral illness or false negative test results in diagnostic tests could result in transmission of infection from the freshwater hatcheries. Hotspot facilities that often are crucial for introducing and spreading infectious diseases and maintaining endemic diseases within the population were absent in the current Atlantic salmon transfer networks. The absence of a positive correlation (facilities with high in-degrees did not have high out-degrees or vice-versa) between in-degree and out-degree supported the absence of hotspots in the Atlantic salmon transfer networks.

The largest strongly connected component (LSCC) of Atlantic salmon transfer networks was consistently small (≤5), which, along with the presence of both disassortative mixing and negative correlation between the in-and out-degree and between ingoing and outgoing infection chain of facilities, rare occurrence of high betweenness and reach, and the identification of freshwater hatcheries as possible superspreaders indicate that Atlantic salmon transfer networks in BC, Canada, are resilient to epidemic spread. However, Atlantic salmon movements from marine netpen sites were randomly inspected typically once a year, and the maximum out-degree from a marine netpen site was 10, which still indicates some risk for spread of infectious diseases, specifically through fish transfers between marine netpen sites. Furthermore, three transfers from marine netpen sites to freshwater hatcheries were recorded. Since marine netpen sites may also contribute to infection dissemination, it is important for facilities to have health protocols when receiving Atlantic salmon from marine netpen sites. For instance, it is necessary to ensure that shipments to a specific facility always come from locations with better or equal health conditions.

Although static networks are easier to analyze, it is important to evaluate how accurately they reflect the underlying temporal dynamics of real-world contact networks. To investigate this, we estimated the causal fidelity of each Atlantic salmon transmission network (39). The analysis revealed high causal fidelity values (>0.80) across Atlantic salmon transfer networks, indicating that static representations provided a reasonable approximation of the network’s overall connectivity. However, causal error estimates suggested that static networks may overestimate potential disease outbreak size by 5 to 23%. This highlights that, while static networks are useful, static networks have limitations when assessing transmission risk. It is also important to note that these estimates were based on a one-day temporal resolution, which may not reflect the actual period of disease transmission, often spanning several weeks or months. A longer temporal window would likely reduce causal error.

The salmonid transfer network in England and Wales (42) revealed that LWCC consisted of 98% of nodes while 94% of the total nodes comprised the network’s maximum reach. The current studied biennial networks had 48 to 97% of nodes in LWCC and 27.40 to 53.03% of nodes in maximum reach, which was less than in the previously explored network in England and Wales (42). To predict the greatest possible spread of epidemics without any intervention, one technique is the LWCC approach (39). Percolation analysis was conducted to simulate the impact of targeted interventions (restriction of trade, vaccination, fallowing, culling, or testing more fish) on the size of LWCC. The findings suggested that targeted removal of highly central nodes based on the outgoing infection chain, outdegree, and total degree value rather than random would be effective in the reduction of LWCC. About 75% of the size of LWCC would possibly be reduced by just taking intervention in 15–20% of the facilities. Since 2019, the targeted removal of nodes appears to be more effective, coinciding with shifts in the demographics of the industry. As freshwater hatcheries were highly central in all the studied networks, just by taking intervention in these facility types, the size of LWCC could be reduced significantly (30 to 80%). However, it would take a large number of marine netpen sites to be removed from the network to reduce the size of LWCC by 75%. Therefore, it would be suggested that alternative disease risk mitigation measures like increased biosecurity, quarantine, emergency vaccination, and testing more fish in freshwater hatcheries would tremendously resist disease epidemics in the BC Atlantic salmon industry.

Community structure shapes disease dynamics, and understanding these structures is a useful epidemiological tool for informing decisions regarding delineating AMUs (54). Community structures support the formulation of surveillance plans that are targeted regionally, as well as the planning, development, and execution of control and eradication initiatives (55, 56). Two or three communities were consistently identified using community detection algorithms in each of the 4 biennial and overall AMU-level networks analyzed, indicating that salmon movements between different AMUs were persistent across the studied time frames. This persistence may have resulted from Atlantic salmon being regularly scheduled to move between designated AMUs at various stages of their life cycles. Furthermore, in the AMU-level biennial network (2021–22) or overall network that had 3 communities, Barkley Sound was the only member of a community. Some AMUs were constantly in the same community in all the biennial networks and overall network. Alternatively, given the fluctuating number of communities in various biannual networks and the periodic turnover of community members, zoning based on facility-level networks would be challenging. For the purpose of improving the sustainability and security of the Atlantic salmon industry, 2 practical zoning techniques can be considered. Reorganizing the movement patterns of the industry to function inside the designated zone is one possibility. An additional strategy would be to break up “risky” linkages between zones by stepping up biosecurity protocols and monitoring the facilities and links that connect them. These actions might be useful in stopping the transmission of infections between far-off locations (45).

This study generated a static temporal network from retrospective data of fish movements, which typically represents the worst-case situation and only provides speculative inference on the potential disease spread. We could not consider other essential transmission routes like shared vehicles, vessels, equipment, and employees, or proximity to other infected sites that contribute significantly to the entry and spread of infectious diseases. Furthermore, the dynamics of disease transmission may be overestimated, as the analysis accounted only for the structural dimension of direct contact, without integrating behavioral aspects that influence real-world disease spread. The data we used did not mention the exact date of Atlantic salmon movement; which is also a limitation. Instead, the available data included transfer license start and end date, a three-month window, and fish were allowed to move any day within that period. Moreover, we assumed all the registered transfers occurred during the licensed period, which in some cases may not have occurred. However, we find insights from this study would be useful despite the imperfect data, and we also think it’s important to identify the database’s strengths and flaws prior to an outbreak rather than after one has occurred. Insights from this study will enable prompt action to prevent and contain the development of an epidemic at its inception. Moreover, it will not only improve monitoring and control during an outbreak but also help develop preventative measures like mobility limitations and targeted vaccination of susceptible fish to keep diseases out of the supply chain. Building on this work, future studies should consider simulating the spread of specific viral or bacterial pathogens through a network-based model to better understand transmission dynamics of such diseases from freshwater hatcheries to marine netpen sites, as well as within and between marine netpen sites.

## 5 Conclusion

Live fish movement represents one of the most important pathways for pathogen spread, and understanding the pattern of movements can aid in assessment of such risk. Our analysis of fish movement in BC identified freshwater hatcheries, which were connected with many marine netpen sites, potentially acting as superspreaders. However, regulations imposed by DFO on fish transfers from freshwater hatcheries (including health inspections, diagnostic tests etc.), and in the absence of identified hotspots, the risk of spread of infectious pathogens attributable to live Atlantic salmon movements in the BC Atlantic salmon industry is minimal. Additionally, in the event of an infectious disease incursion, focused surveillance and mitigation (if necessary) efforts could be targeted to facilities close to high-centrality network nodes. However, our network-based approach highlights structural vulnerabilities and identifies facilities with high centrality that may warrant targeted surveillance and contingency planning, it does not incorporate pathogen-specific characteristics or other important transmission pathways (such as vessel, personnel, or equipment movement between facilities). These findings provide a structural perspective on how diseases could spread through fish movements, but they should not be interpreted as a precise prediction of disease risk. To fully understand the potential for disease transmission, additional information, such as specific pathogen characteristics and other on-facility practices, would need to be considered.

## Data Availability

The datasets presented in this study can be found in online repositories. The names of the repository/repositories and accession number(s) can be found at: https://search.open.canada.ca/data/.

## Footnotes

^1^ https://open.canada.ca
