# Supplementary material for: Network analysis of farmed Atlantic salmon movements in British Columbia, Canada
Source: Front Vet Sci. 2025 Jun 18;12:1568484. doi: 10.3389/fvets.2025.1568484 (PMC12218256; doi:10.3389/fvets.2025.1568484)
Supplement: Supplementary file 1 [file Data_Sheet_1.docx]

Supplementary Material

**Supplementary Table 1**: Number of different facility types present in different biennial networks and overall networks (FH- Freshwater hatchery, MN- Marine netpen site).

| Facility type | 2015-16 | 2017-18 | 2019-20 | 2021-22 | Overall |
| --- | --- | --- | --- | --- | --- |
| FH | 11 | 9 | 9 | 9 | 11 |
| MN | 62 | 76 | 69 | 57 | 88 |

**Supplementary Table 2**: Movement of Atlantic Salmon at different life stages in different biennial network windows in British Columbia, Canada.

|  | Year | Farms (Nodes) | Edges (Unique link) | Total link |
| --- | --- | --- | --- | --- |
| **Types of Fish** | | | |  |
| Atlantic Salmon | 2015-16 | 73 | 118 | 177 |
|  | 2017-18 | 85 | 153 | 206 |
|  | 2019-20 | 78 | 150 | 195 |
|  | 2021-22 | 66 | 165 | 234 |
|  | Overall | 99 | 350 | 812 |
| **Life stages** | | | | |
| Fry | 2015-16 | 0 | 0 | 0 |
|  | 2017-18 | 3 | 2 | 2 |
|  | 2019-20 | 0 | 0 | 0 |
|  | 2021-22 | 4 | 3 | 3 |
|  | Overall | 4 | 3 | 5 |
| Smolt | 2015-16 | 71 | 114 | 172 |
|  | 2017-18 | 82 | 146 | 194 |
|  | 2019-20 | 76 | 145 | 189 |
|  | 2021-22 | 65 | 153 | 216 |
|  | Overall | 98 | 329 | 771 |
| Brood | 2015-16 | 0 | 0 | 0 |
|  | 2017-18 | 3 | 2 | 2 |
|  | 2019-20 | 4 | 4 | 4 |
|  | 2021-22 | 4 | 3 | 3 |
|  | Overall | 11 | 9 | 9 |
| Adult | 2015-16 | 6 | 4 | 5 |
|  | 2017-18 | 10 | 6 | 8 |
|  | 2019-20 | 2 | 1 | 2 |
|  | 2021-22 | 9 | 8 | 12 |
|  | Overall | 20 | 17 | 27 |

**Supplementary Table 3:** Atlantic salmon movement between different facility types in different biennial network windows.

| Movement | 2015-16 | 2017-18 | 2019-20 | 2021-22 | Overall |
| --- | --- | --- | --- | --- | --- |
| FH to FH | 0 | 4 | 5 | 5 | 14 |
| FH to MN | 134 | 146 | 152 | 177 | 609 |
| MN to FH | 0 | 0 | 0 | 3 | 3 |
| MN to MN | 43 | 56 | 38 | 49 | 186 |

**Supplementary Table 4:** Number of SCC and WCC and length of 2^nd^ largest SCC and WCC in different network snapshots.

|  | 2015-16 | 2017-18 | 2019-20 | 2021-22 | Overall |
| --- | --- | --- | --- | --- | --- |
| # SCC | 73 | 85 | 78 | 61 | 88 |
| Size of 2^nd^ largest SCC | 1 | 1 | 1 | 2 | 4 |
| # WCC | 4 | 3 | 3 | 2 | 2 |
| Size of 2nd largest WCC | 33 | 41 | 35 | 2 | 2 |

**Supplementary Table 5:** Clustering coefficient and average path length of facility-level networks with random networks created using the Erdos-Renyi model (Small world topology) and Small-world-ness (S) of Atlantic salmon transfer networks.

|  | 2015-16 | 2017-18 | 2019-20 | 2021-22 | Overall |
| --- | --- | --- | --- | --- | --- |
| Average path length | 3.61 | 3.49 | 3.29 | 2.72 | 2.54 |
| Clustering coefficients | 0.04 | 0.04 | 0.05 | 0.08 | 0.07 |
| Small-world-ness (S) | S= 6.34 | S=4.97 | S=5.44 | S=2.99 | S=4.26 |

| Networks | Causal fidelity | Causal error |
| --- | --- | --- |
| 2015-16 | 0.95 | 1.05 |
| 2017-18 | 0.92 | 1.09 |
| 2019-20 | 0.94 | 1.06 |
| 2021-22 | 0.81 | 1.23 |
| Overall | 0.83 | 1.20 |

**Supplementary Table 6:** The assessment of causal fidelity and causal error

**Supplementary Table 7:** Distribution (n, %) of facilities situated in different aquaculture management units (AMU) during the study period.

| Aquaculture Management Unit (AMU) | 2015-16 | 2017-18 | 2019-20 | 2021-22 | Overall |
| --- | --- | --- | --- | --- | --- |
| Broughton Archipelago(BA) | 15(20.55) | 20(23.53) | 17(21.9) | 14(21.21) | 21 (21.21) |
| Clayoquot Sound(CS) | 12(16.44) | 12(14.12) | 12((15.68) | 12(18.18) | 17 (17.17) |
| Discovery Islands(DI) | 13(17.80) | 16(18.82) | 16(20.51) | 6(9.09) | 16 (16.16) |
| Nootka Sound(NS) | 5(6.84) | 7(8.24) | 6(7.69) | 6(9.09) | 7(7.07) |
| Sunshine Coast(SC) | 7(9.59) | 6(7.06) | 6(7.69) | 6(9.09) | 8(8.08) |
| Salish Gulf Islands(SGI) | 3(4.11) | 2(2.35) | 2(2.56) | 2(3.03) | 3(3.03) |
| Port Hardy(PH) | 7(9.59) | 10(11.76) | 8(10.26) | 6(9.09) | 10 (10.10) |
| Central Coast(CC) | 6(8.22) | 5(5.88) | 4(5.13) | 5(7.58) | 8 (8.08) |
| Esperanza Inlet(EI) | 2(2.74) | 3(3.53) | 3(3.85) | 4(6.06) | 4 (4.04) |
| Quatsino Sound(QS) | 2(2.74) | 4(4.71) | 4(5.13) | 4(6.06) | 4 (4.04) |
| Barkley Sound(BS) | 1(1.37) | 0(0.00) | 0(0.00) | 1(1.52) | 1 (1.01) |


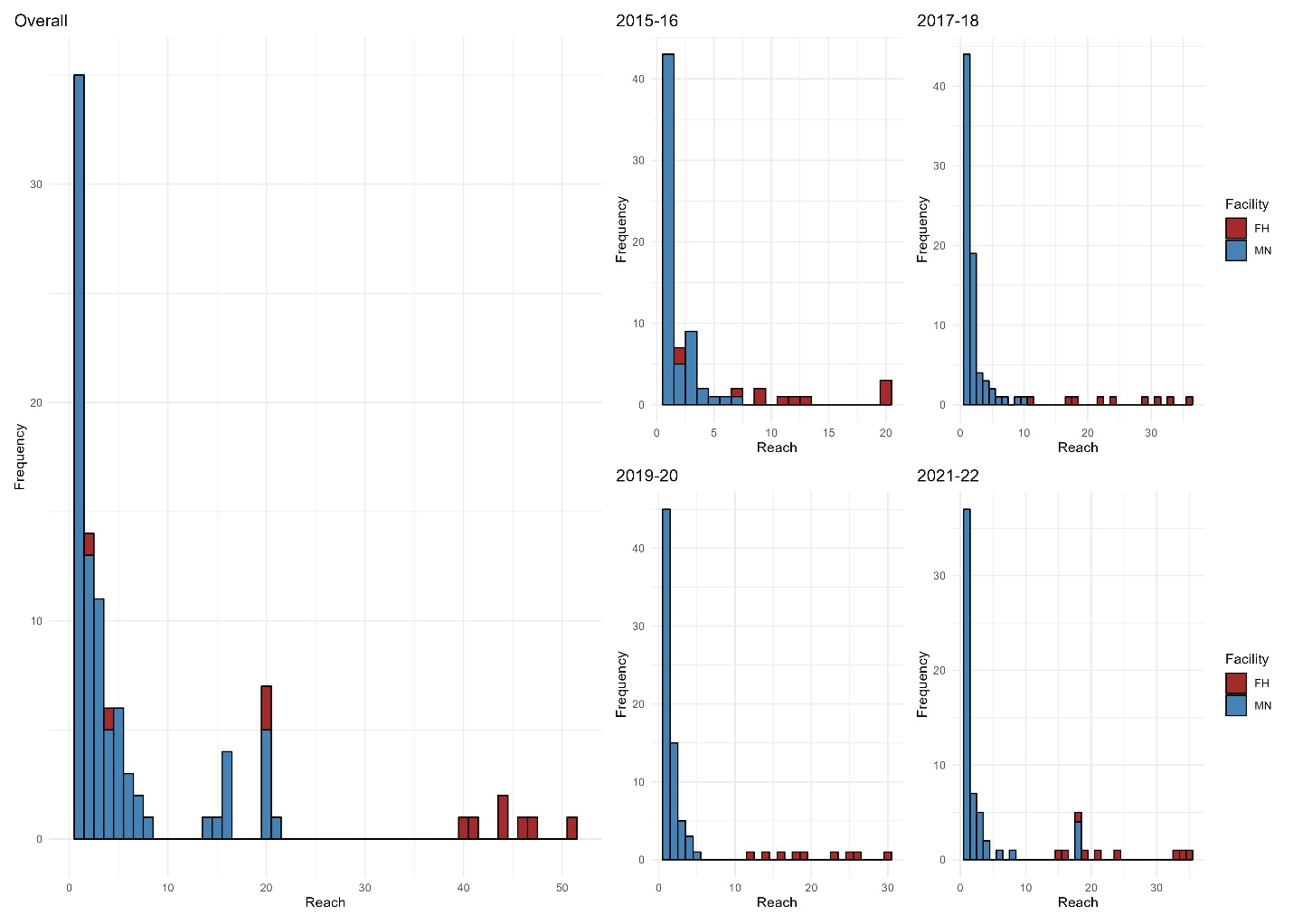


**Supplementary Figure 1:** Node reach of different facility types. (FH- Freshwater hatchery, MN- Marine netpen site).


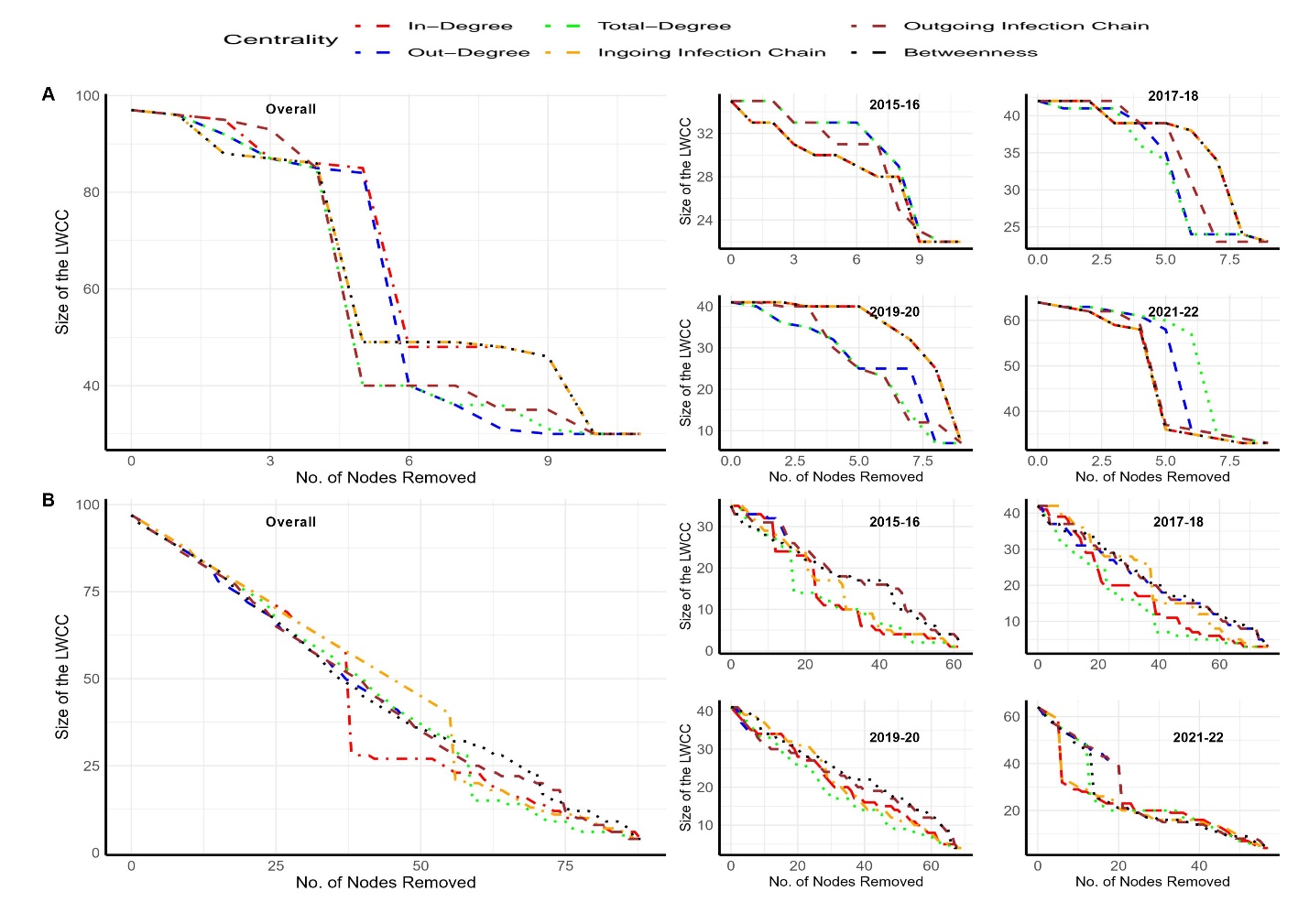


**Supplementary Figure 2:** Effectiveness of targeted removal of A. FH facilities B. MN facilities over the size of LWCC in different biennial and overall networks.


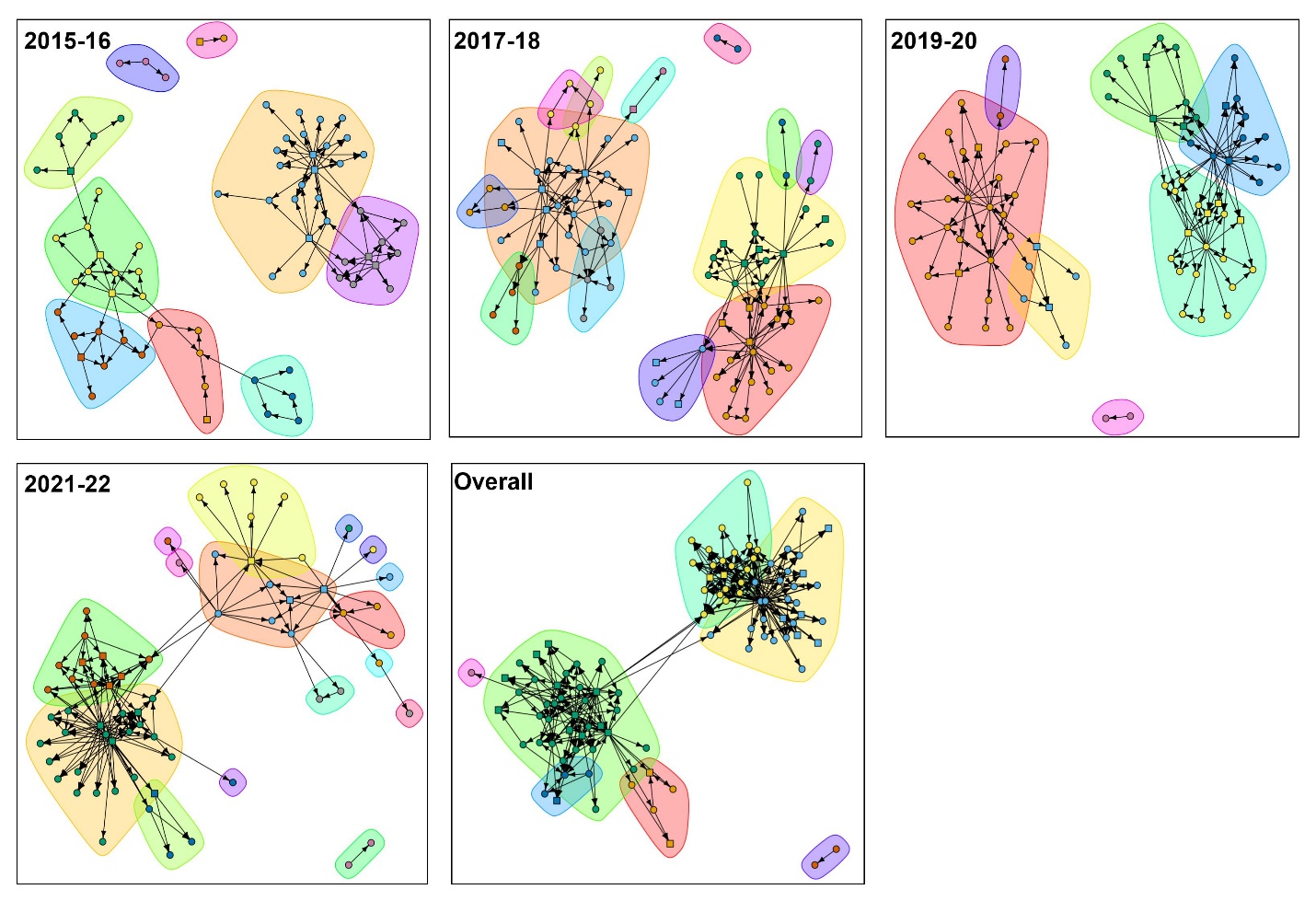


**Supplementary Figure 3**: Trade communities in different facility-level Atlantic salmon networks. Each shaded color represents a specific community within the transfer network. Arrows indicate the direction of transfers between different facilities.
